# A Gambian Infant with Fever and an Unexpected Blood Film

**DOI:** 10.1371/journal.pmed.0030355

**Published:** 2006-09-26

**Authors:** Stephen Howie, Malcolm Guy, Louise Fleming, Wendi Bailey, Harry Noyes, Joseph Axel Faye, Jacques Pepin, Brian Greenwood, Hilton Whittle, David Molyneux, Tumani Corrah

## Abstract

The authors describe the differential diagnosis, investigation, and management of a two-month-old infant with edema, malnutrition, and fever.

## DESCRIPTION of CASE

The patient, a two-month-old Gambian infant, was one of twins born in November 2003. In the latter part of her pregnancy, the patient's mother went to stay with her family in southern Senegal, where she delivered at uncertain gestation in a village health centre. The patient, a 1.75-kg girl, and her sibling, a 2.4-kg boy, were both born alive. Soon after delivery the twins' mother died. The cause of her death is not known.

The twins were brought from Senegal to Gambia, where the second twin died, again of an unknown cause. The patient was cared for by an aunt who, being unable to lactate, attempted to feed her with an infant formula intended for use beyond six months of age, though the limited financial means of the family made this difficult. The patient was admitted to the Royal Victoria Teaching Hospital in Gambia at seven weeks of age with a history of fever and generalised oedema.

**Figure 1:**
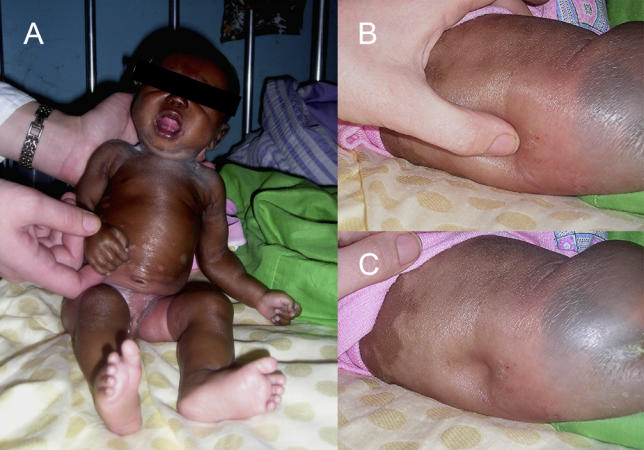
Photographs of the Patient Soon after Admission, Showing Generalised Oedema (A) shows the frontal view. (B) and (C) demonstrate oedema of the back.

On examination the patient looked unwell, was grossly oedematous, and weighed 2.3 kg (Figure 1). She was alert and had no evidence of neurological abnormality. A low-grade pyrexia was present, but there were no other abnormal physical findings.

### What Are the Likely Causes of This Presentation and Which Tests Would You Perform?

For any sick young infant in any setting, the possibility of infection must be foremost in the clinician's mind. Neonates and young infants are particularly susceptible to infection, the more so when they are low birth weight and suffering from malnutrition, as must be suspected in a motherless child in this setting. Bacterial infections top the list, with group B streptococcus, gram-negative organisms (such as *Escherichia coli*), pneumococcus, *Haemophilus influenzae* type b, and *Staphylococcus aureus* being common pathogens. A systematic search for a focus of infection is needed because young infants localise infections poorly and present with non-specific symptoms and signs. While respiratory distress may indicate pneumonia, a swollen joint may indicate septic arthritis, and a bulging fontanelle, seizures, and depressed level of consciousness may indicate meningitis, these signs cannot be relied upon. A sepsis work-up in any sick young infant should include, regardless of signs, microscopy and culture of urine and cerebrospinal fluid (CSF), blood culture, and a chest radiograph as well as swabs of suspect lesions.

In endemic countries malaria is uncommon in very young infants, in part because of the presence of maternal antibodies and high levels of fetal haemoglobin, but nevertheless a blood film is appropriate to rule out malaria.

HIV infection must be considered in this sick infant whose mother and sibling have died. Without intervention, mother-to-child transmission of HIV-1 occurs during pregnancy and delivery in 15–30 percent of infants and via breast-milk in 10–20 percent [1]. Perinatally acquired HIV progresses rapidly in up to 25 percent of infants and usually presents with non-specific features. Malnutrition, diarrhoea, pneumonia, dermatitis, lymphadenopathy, hepatosplenomegaly, oral candidiasis, and parotitis are among the commoner features. The diagnosis of HIV infection in infants is complicated by the passive transfer of maternal anti-HIV antibodies. Antibody-based testing cannot reliably confirm infection until the infant is 18 months old. However, polymerase chain reaction testing to detect the presence of HIV DNA can confirm infection in the early stages.

The differential diagnosis of pyrexia in an infant in the tropics includes a range of other infections, including tuberculosis and salmonellosis, the likelihood of which will be influenced by local epidemiology and specific features of the presentation.

In this case, oedema was the major feature that accompanied the pyrexia and it requires explanation as it is unusual in young infants. Heart failure is a cause of generalised oedema but will be accompanied by other signs such as tachypnoea, cardiomegaly, hepatomegaly, and often a heart murmur, all of which were absent in this case. Renal causes such as nephrotic syndrome are unlikely. In this setting, an infant whose mother has died is very much at risk of malnutrition. Kwashiorkor, a less common manifestation of malnutrition in young infants than marasmus, is one possible explanation for the oedema in this case, though the severity and transience of it would be unusual. Basic anthropometry (weight, length/height, weight-for-length/height *z*-score) should be routine in the assessment of sick children, though anthropometry may not indicate malnutrition when the patient has oedema (as in this case where the *z*-score was above the median). Transient oedema can also be a feature of African trypanosomiasis.

The child was treated for malnutrition with appropriate nutritional support and broad-spectrum antibiotics. The sepsis work-up was negative, but on a routine thick blood film, which was negative for malaria parasites, the microscopist was surprised to see numerous extracellular flagellate parasites, identified by a senior colleague as trypanosomes. The child was referred to the Medical Research Council Laboratories hospital in Gambia for further management. Tests for HIV-1 and HIV-2, both antibodies (Murex HIV-1.2.0, Abbott-Murex) and polymerase chain reaction, were negative.

### What Are the Most Important Complications of Human African Trypanosomiasis? How Can the Diagnosis of Human African Trypanosomiasis Be Confirmed?

**Figure 2:**
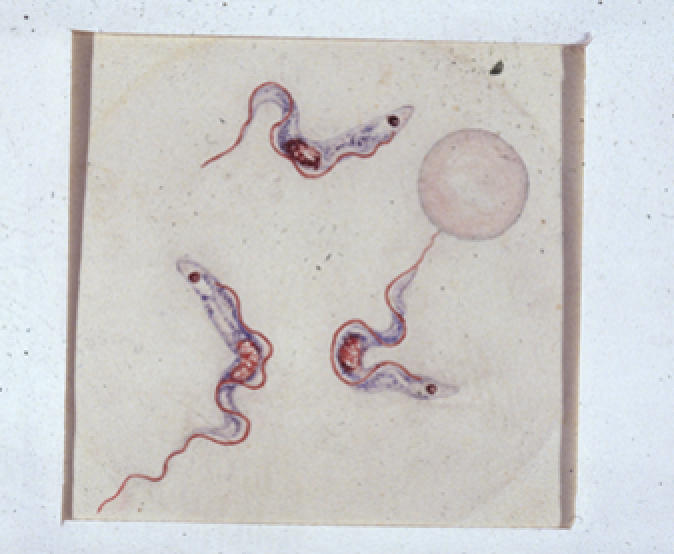
Watercolour of *T. b. gambiense* from the Original Description of the First Patient by J. E. Dutton, Gambia, 1902.

Human African Trypanosomiasis (HAT), also known as sleeping sickness, is caused by *Trypanosoma brucei rhodesiense* (in East and Southern Africa) or *Trypanosoma brucei gambiense* (in West and Central Africa). Infection with *T. b. gambiense* was first described by Dutton in 1902 (Figure 2) in Gambia in a riverboat captain by the name of Kelly. Although it has re-emerged in several countries in sub-Saharan Africa, it has not been seen in Gambia, Senegal, or Guinea-Bissau for more than 20 years [2].

Disease due to *T. b. rhodesiense* progresses rapidly over weeks, while disease due to *T. b. gambiense* tends to progress more slowly over months. In either case, infection leads to invasion of the central nervous system (CNS) and death if untreated. The two subspecies are morphologically indistinguishable. Fortunately, from the point of view of diagnosis, their geographical distribution does not overlap, though there is now concern about geographical convergence in Uganda [3].

The diagnosis of HAT caused by *T. b. gambiense* presents different challenges from that caused by *T. b. rhodesiense*, which is usually directly detectable in the blood. A stepwise approach to the diagnosis of *T. b. gambiense* starts with a serological test such as the card agglutination test for trypanosomiasis (CATT), a field diagnostic test of high sensitivity but lower specificity (due to cross-reactivity with animal trypanosomes), followed by parasitological diagnosis from blood or lymph samples. In *T. b. gambiense* infections, detection of trypanosomes in blood is difficult, given the low-level and intermittent parasitaemia. In both cases, detection of parasites in blood or lymph node samples or clinical suspicion alone mandates examination of the CSF to determine the presence of CNS disease (late-stage HAT). Parasites can easily be missed on microscopy of a simple wet preparation of CSF, and sensitivity is increased by examining a doubly centrifuged specimen. The presence of CSF trypanosomes or a CSF white cell count of >5 cells per mm^3^ is diagnostic of late-stage trypanosomiasis.

In this case a second blood film at the Medical Research Council Laboratories hospital confirmed the presence of numerous trypanosomes. A CSF sample was blood-stained and showed trypanosomes on a wet preparation. A CSF sample repeated five days later, before any specific treatment was given, showed again the presence of occasional trypanosomes. This specimen was not blood-stained and showed no pleocytosis. The finding of live trypanosomes in two CSF samples taken five days apart suggests that the CNS had been invaded, although no neurological abnormalities were found. It is not certain whether this invasion was spontaneous or a result of seeding from the first lumbar puncture.

### What Treatment Is Indicated for HAT?

In the absence of CNS involvement (i.e., in early-stage HAT), pentamidine is the drug of choice for infection with *T. b. gambiense* while suramin is indicated for *T. b. rhodesiense*. Pentamidine is associated with cure rates above 90 percent in *T. b. gambiense* infection [4]. Hypotension is the chief immediate adverse drug reaction, occurring more frequently when administered intravenously than intramuscularly, but pentamidine also causes potentially severe adverse effects such as hypoglycaemia, hypocalcaemia, renal failure, neutropenia, and ventricular arrhythmia, all of which make it an unattractive option for treating an infant. In *T. b. rhodesiense* infection, treatment failures with pentamidine are common, making suramin the drug of choice for early-stage disease.

Both pentamidine and suramin penetrate CSF poorly; therefore, given the presence of CSF trypanonosomes in this case, the alternatives for the treatment of presumptive *T. b. gambiense* infection were eflornithine or melarsoprol. Eflornithine is a trypanostatic drug associated with bone marrow suppression in up to half of recipients, though this usually resolves at the end of treatment. Melarsoprol is a trivalent arsenical drug that is highly trypanocidal, but also causes a potentially fatal encephalopathy in five to ten percent of patients. Eflornithine needs to be given six-hourly intravenously for 14 days, which is impractical in a developing-world setting, and the dose for an infant is not well established. Melarsoprol was the only practical option; therefore, despite its potential toxicity, it was given at a dose of 2.2 mg/kg daily intravenously for ten days, along with prednisolone (1 mg/kg daily) to reduce the risk of melarsoprol-induced encephalopathy [5,6].

### Was This Case *T. b. gambiense* HAT?

**Figure 3:**
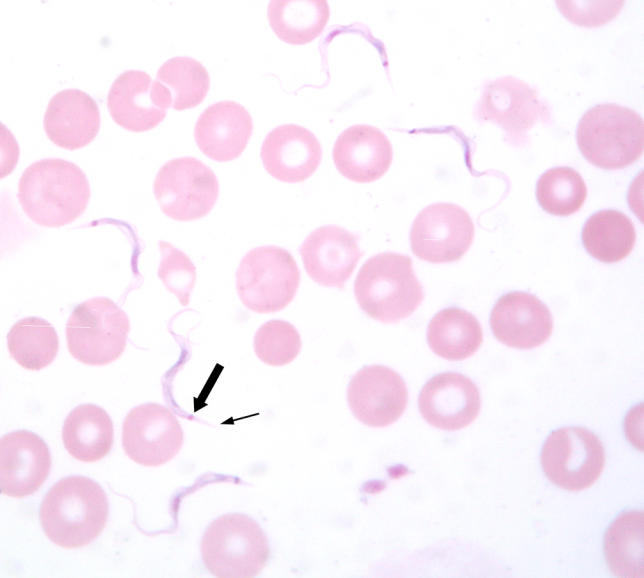
Thin Blood Film of the Patient Showing Five Trypanosomes and Several Red Blood Cells Arrows indicate the pointed posterior segment (small arrow) and the large kinetoplast (large arrow) that distinguish these from *T. b. gambiense*.

**Figure 4:**
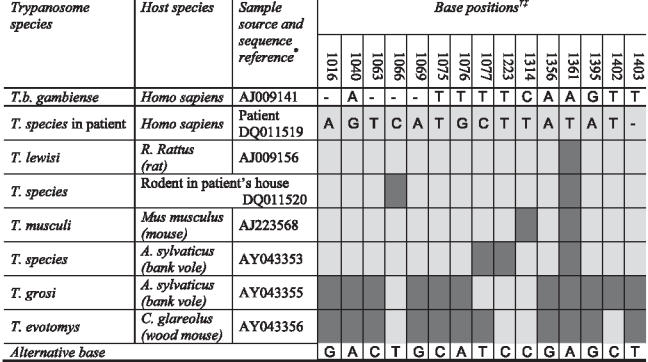
Genotypes of the Parasite Isolated from the Patient, the Parasite Isolated from a Rodent Captured in the Patient's Home, *T. (Herpetosoma)* Species and *T. b. gambiense* at Positions on the 18S Ribosome that Are Polymorphic within the Subgenus *T. (Herpetosoma)* *, alphanumeric codes represent GenBank numbers for reference samples. †, numbers of base positions are relative to the *T. lewisi* reference sequence AJ009156. ‡, the base at all positions is recorded for *T. b. gambiense* and the patient's parasite, while the base in other strains is indicated by light shading where it is identical to the base of the patient's parasite and by dark shading where it matches the alternative base.

Confirmed infection with a trypanosome should be regarded as HAT until proven otherwise. In this case, the thin blood film done to examine the morphology of the trypanosome raised a doubt that was not resolved one way or the other until after the acute management of the patient. The appearance of the parasites was not typical of *T. b. gambiense* (Figure 2), but was typical of rodent trypanosomes of the subgenus *T. (Herpetosoma)* (Figure 3). Antibodies to *T. b. gambiense* were not detected in blood or CSF by fluorescent antibody testing. Sequencing of part of the 18S ribosomal RNA confirmed a genotype quite distinct from that of *T. b. gambiense* but very similar to that of the *T. (Herpetosoma)* group, particularly *T. lewisi*, from which it differed by just one base (Figure 4) [7]. Molecular methods may become increasingly important in the diagnosis and management of HAT if there is, as feared, convergence of the geographical ranges of *T. b. gambiense* and *T. b. rhodesiense* [3].

### Progress

The patient's oedema resolved without specific treatment over the course of a week, and she remained clinically stable until therapy with melarsoprol was started. A repeat blood film the day before starting treatment, 15 days after initial presentation, showed a persistent high density of trypanosomes. Three days into treatment a blood film showed no evidence of trypanosomes. Treatment was well tolerated: apart from a temperature of up to 38 °C early in the course of treatment, a well-described phenomenon, the patient remained well and had no signs of encephalopathy [8]. Repeat blood film and CSF samples taken at the completion of treatment and ten months later showed no evidence of parasites, and there were no white cells in the CSF. Follow-up to 13 months of age confirmed that the child was making normal developmental progress.

Visits were made to the family homes in Gambia and Senegal. The caregiver's house in rural Gambia was reportedly infested with rodents, which was confirmed by the finding of rodent droppings on the bed where the patient slept and a dead rodent on the porch. The mother's home also reportedly had a rodent problem, but there was no report of this in her family's home in Senegal.

Two live rodents of unidentified species were trapped at the caregiver's home. Examination of a blood film revealed trypanosomes morphologically identical to those seen in the patient. Genotyping of a part of the 18S ribosome revealed a sequence differing by two bases from that of the patient's parasite (Figure 4), which may well represent variation within the species rather than a different species [9]. A blood film of the caregiver showed no evidence of trypanosomes.

## DISCUSSION

This unique case has provided the opportunity to review approaches to the unwell young infant in general and the management of suspected HAT in particular. To our knowledge, this is the first documented case of an infection by trypanosomes of the subgenus *T. (Herpetosoma)* in humans in Africa, and the first case anywhere in which this parasite has been able to infect the CNS and survive. As such, it offers the opportunity to review what is known of this parasite, and to consider the environmental, host-related, and organism-related factors that may have resulted in the child's illness.

The subgenus *T. (Herpetosoma)* comprises at least 45 morphologically identical species that infect rodents throughout the world [10,11]. *T. lewisi*, the archetype of the subgenus, is a parasite of the genus *Rattus* and is transmitted via the excreta of fleas. In West Africa there are records of *T. (Herpetosoma)* infection in members of the genera *Cricetomys* (Gambian giant rat) and *Mastomys* (multimammate rat) as well as in *Rattus* itself [12].

There are, to our knowledge, only three reported cases of human infection with a *T. lewisi*–like parasite, though in the absence of expert microscopy other cases might be misdiagnosed. All reported cases occurred in Asia, and all were reliant on morphology for species identification.

The first patient, described in 1933, was a four-month-old Malaysian child who lived in a rat-infested dwelling [13]. The child presented with fever and lassitude associated with a heavy parasitaemia with *T. lewisi*. No CSF investigation was reported. The parasitaemia and accompanying symptoms resolved without specific treatment after five days. Blood films from other family members were all negative. One rat caught in the dwelling had a heavy infection with a morphologically identical parasite. This patient was followed up years later and found to have no trace of the parasite in blood or CSF [14].

The second report (of two cases) was from India in 1974 [15]. An adult couple who lived in a rat-infested village both presented with fever and one had malaise. Both were found to have heavy *T. lewisi* parasitaemia. Symptoms resolved without specific treatment after two to three days. Repeat blood films taken eight weeks later were negative.

In the present case, the parasitaemia persisted unabated for more than two weeks until specific therapy was started, unlike the Malaysian infant reported by Johnson [13]. Neither was oedema a feature of previous cases, though it is characteristic of HAT and it is conceivable that *T. lewisi* infection caused the oedema in this case.

It is unclear in this case whether infection was acquired antenatally or postnatally. Congenital infections with *T. b. gambiense* and *T. b. rhodesiense* have been documented [10]. The usual duration of the incubation period of *T. b. gambiense* is in the order of months, whereas *T. (Herpetosoma)* infections develop detectable parasitaemias typically within seven to 14 days of inoculation in rodents. There is no evidence that *T. (Herpetosoma)* trypanosomes can be transmitted transplacentally in rodents [16]. This suggests that in our patient, transmission occurred after delivery through exposure to the excreta of infected fleas in the environment in which the child was living.

Host factors are likely to have been important in this case: the relative immune vulnerability of early infancy together with malnutrition probably increased this child's susceptibility to this unusual infection. Humans naturally resist infection by animal trypanosomes such as *T. brucei*, *T. lewisi*, *T. congolense*, and *T. vivax*. What drives this resistance of human serum to animal trypanosomes has been debated, but recent research suggests that for *T. brucei* it is an apolipoprotein, either a haptoglobin-related protein or apolipoprotein L-I [17,18]. Whether similar factors are important in controlling infections with *T. lewisi* is unknown.

Organism factors may also have played a part in this case. It is possible that this patient's parasite has become better adapted to human infection than its forebears, though we cannot say this with certainty. Virulence varies between subspecies of *T. brucei*: *T. b. rhodesiense* is more virulent than *T. b. gambiense*, arguably killing its host too rapidly for its own interest. This is related to the serum resistance–associated *SRA* gene, which is now used for the molecular epidemiology of *T. b. rhodesiense* infections and, as its name implies, is associated with resistance to human serum in vitro and in vivo [3]. The continuing adaptation of organisms to human infection will be influenced by changing host factors, and in this respect there is concern that the HIV pandemic might create a new window of opportunity for organisms to make this transition [19].

### Conclusion

The lessons from this learning forum are several. Firstly, infection is a major cause of mortality in young infants and must be managed diligently with a thorough search for its cause and the institution of appropriate treatment. Secondly, HAT remains a potentially fatal disease that presents challenges in the difficulty of its diagnosis and in the toxicity of its treatments. Additionally, given the right combination of environmental, host-related, and organism-related factors, a normally harmless organism can emerge as a human pathogen. This first report of invasive disease from *T. lewisi* shows that this parasite cannot be dismissed as a harmless cause for false positives in the work-up of suspected cases of HAT. We believe that on current evidence this infection should be treated when diagnosed. However, the data on which to make this decision are limited, so in asymptomatic confirmed cases without CNS invasion a case can be made for careful follow-up without treatment. Nevertheless, the final message must be that if there is any evidence of invasive disease, any doubt concerning the identity of the parasite, or any concern regarding the effectiveness of follow-up, standard treatment for HAT should not be delayed.

**Learning Points**

- Infection is a major cause of mortality in young infants and requires a thorough search for its cause and the institution of appropriate treatment.
- HAT remains a potentially fatal disease that can be challenging to diagnose.
- Treatments for HAT are associated with toxicities (e.g., pentamidine can cause hypotension, eflornithine can cause bone marrow suppression, melarsoprol can cause encephalopathy).
- Given the right combination of environmental, host-related, and organism-related factors, a normally harmless organism can emerge as a human pathogen.
- If in doubt, treat human infection with non-typical trypanosomes as HAT.

## Abbreviations

CATT: card agglutination test for trypanosomiasis
CNS: central nervous system
CSF: cerebrospinal fluid
HAT: Human African Trypanosomiasis
